# Thyrotoxic periodic paralysis presenting in an African-American teenage male: case report

**DOI:** 10.1186/s13633-020-00077-3

**Published:** 2020-04-24

**Authors:** Joshua Glass, Jennifer Osipoff

**Affiliations:** 1 Department of Pediatrics, Stony Brook Children’s Hospital, 101 Nicolls Road, HSC-T11, Room 040, Stony Brook, NY 11794 USA; 2 Division of Endocrinology, Department of Pediatrics, Stony Brook Children’s Hospital, 101 Nicolls Road, HSC-T11, Room 040, Stony Brook, NY 11794 USA

**Keywords:** Thyrotoxic periodic paralysis, Hyperthyroidism, Thyrotoxicosis, Hypokalemia, Adolescent

## Abstract

**Background:**

Thyrotoxic periodic paralysis is a rare complication of hyperthyroidism and is associated with hypokalemia and muscle paralysis. This condition is most commonly seen in Asian men.

**Case presentation:**

We report on a 14-year-old African American male with Graves’ disease and intermittent asthma who presented with bilateral leg weakness. The patient demonstrated signs of thyrotoxicosis and laboratory evaluation revealed hypokalemia and hyperthyroidism. Following the administration of potassium supplementation clinical status improved and the patient was discharged home on a high dose of methimazole and propranolol. At a 6-month follow up visit, he was found to be clinically euthyroid and demonstrated no signs of hyperthyroidism or muscle weakness.

**Conclusion:**

Children presenting with weakness and hypokalemia should be investigated for thyroid dysfunction. Correction of hypokalemia improves acute presentation, but the patient will remain at risk for paralysis until euthyroid state is achieved.

## Background

Hypokalemic thyrotoxic periodic paralysis (HTPP) occurs as a rare complication of hyperthyroidism. It is associated with a significant intracellular shift of potassium and manifests itself clinically by hypokalemia and muscle paralysis.

HTPP is well described in the literature to predominantly affect Asian males [1–4]. Despite a higher incidence of thyrotoxicosis in females (female-to-male ratio of 9:1), HTPP is a condition that is seen mainly in men (male-to-female ratio ranging from 17:1 to 70:1) [5]. The average age of incidence is between 20 to 40 years of age [3]. Cases of HTPP in African-American males are rare [6, 7].

We present a case of hypokalemic thyrotoxic periodic paralysis in an African-American teenage male diagnosed previously with Graves’ disease. The patient’s presentation was likely triggered as a result of poor medication compliance, returning him to a hyperthyroid state. Treatment was complicated secondary to his medical history significant for asthma.

## Case presentation

A 14-year-old African-American male with hyperthyroidism and intermittent asthma from Long Island, New York presented with acute onset of bilateral leg weakness. The patient reported experiencing bilateral leg soreness after swimming 2 days prior to presentation but denied particularly intense activity. The soreness progressed to generalized weakness and pain. Upon wakening to go to the bathroom in the early morning, the patient found himself unable to bear his own weight. The patient otherwise denied recent fevers, shortness of breath, cough, congestion, nausea, vomiting, diarrhea, rash, headaches, or visual disturbances. Recent diet was not ascertained, however the patient reported eating his usual dinner the night prior to the onset of symptoms.

With regards to his past medical history, he was diagnosed with reactive airway disease at 8 months of age. He was well controlled on inhaler therapy with budesonide and albuterol and he had recently required rescue albuterol after experiencing chest tightness while playing football. Three months prior to this presentation he was evaluated for the complaints of fatigue, tremors, palpitations, heat intolerance, difficulty focusing in school, and weight loss without any history of muscle weakness. His physical examination was remarkable for a nontender goiter and proptosis. Subsequent bloodwork revealed a thyroid-stimulating hormone (TSH) level less than 0.01 mIU/L (normal 0.52–5.05 mIU/L) and an elevated T4 level of 30.4 mcg/dL (normal 4.84–10.13 mcg/dL), consistent with the diagnosis of Graves’ disease. After a pediatric endocrinology consultation, he was prescribed methimazole (5 mg every morning, 10 mg every evening) and 25 mg atenolol daily. He was also instructed to avoid physical activity until his thyroid hormones had declined. He reported good medication compliance until approximately 2 weeks prior to current presentation at which point he ran out of medication at home and failed to refill the prescriptions. Family history was only significant for a maternal grandmother with hyperthyroidism.

On presentation to the emergency department the patient’s vital signs were notable for tachycardia (109 bpm) and an elevated blood pressure (154/87 mmHg). Physical examination was remarkable for bilateral lower and upper extremity weakness with lower extremity areflexia and upper extremity hyporeflexia. Initial laboratory tests revealed a potassium level of 2.0 mmol/L (normal 3.4–4.7 mmol/L) and a TSH level less than 0.005 mIU/L. Electrocardiogram demonstrated normal sinus rhythm, no ST elevation, and positive U waves. Initial concern for intracranial hemorrhage or other central nervous system abnormalities were ruled out following normal imaging of the brain, including computed tomography scan and magnetic resonance imaging. The patient was given 40 meq potassium oral replacement therapy, 1 L of normal saline mixed with 20 meq of potassium, and 1 g of magnesium before being transferred to our hospital.

On arrival to the pediatric intensive care unit, the patient appeared comfortable and was answering questions appropriately. His muscle weakness had dramatically improved, however he continued to remain tachycardic (117 bpm) and hypertensive (128/70 mmHg). Muscle power in the proximal right lower extremity was appreciated to be 4/5, while full muscle power was appreciated in all the other extremities. Deep tendon reflexes in bilateral lower and upper extremities were 1+ and 2+ respectively. Cranial nerves II-XII were grossly intact and the rest of the neurologic examination was otherwise unremarkable. A thyroid function panel was obtained and demonstrated a TSH level of less than 0.005 mIU/L, T3 level greater than 651 ng/dL (normal 110.02–184.88 ng/dL), T4 level of 16.8 mcg/dL, and a free T4 level greater than 7 ng/dL (normal 1.03–1.77 ng/dL). Repeat basic metabolic panel revealed an improved potassium level of 4.7 mmol/L and magnesium level of 1.8 mg/dL. The pediatric endocrinology team advised re-initiation of 20 mg methimazole two-times daily and 20 mg propranolol three-times daily. His clinical status rapidly improved and he was discharged home on the second day of admission.

Upon follow-up the patient reported good medication compliance with no further episodes of weakness or worsening of pulmonary symptoms. Because he was asymptomatic, the propranolol was discontinued after 1 month and methimazole dosing was decreased to 10 mg daily after 3 months. Six months after discharge the patient remained clinically euthyroid. His thyroid hormonal profile revealed normalization of T4 (7.6 mcg/dL) however, he continued to have a low TSH (0.02 mIU/L) and an elevated thyroid stimulating immunoglobulin (400%; normal < 140%). He was instructed to continue methimazole 10 mg daily and to obtain repeat thyroid function tests prior to the next appointment in 3 months.

## Discussion

Hypokalemic thyrotoxic periodic paralysis is a challenging diagnosis to make as it is rare in non-Asian populations and has a broad differential diagnosis. Thyrotoxic symptoms may be subtle and overlooked. Alternatively, assessing thyroid function helps to distinguish HTPP from other forms of periodic paralysis. Hypokalemic paralysis can be the result of a transcellular shift of potassium (i.e. thyrotoxic periodic paralysis, familial periodic paralysis), a renal loss of potassium (i.e. diuretic use, primary hyperaldosteronism, renal tubular acidosis), or a gastrointestinal loss of potassium (i.e. celiac disease, infectious diarrhea) [4]. The differential diagnosis for periodic paralysis also includes Guillain-Barre syndrome, transverse myelitis, and acute spinal cord compression; bladder and bowel function remain unaffected in HTPP [1].

HTPP is characterized by hyperthyroidism, muscle paralysis, and hypokalemia without total body potassium deficit [4]. Recent studies suggest that the condition is precipitated by a combination of thyrotoxicosis, environmental factors such as a high carbohydrate load or intense exercise, and an underlying genetic mutation, all of which lead to increased sodium-potassium-adenosine triphosphate (Na/K-ATPase) pump activity [8]. A proposed multifactorial model elucidates Na/K-ATPase pump activity amplification. First, thyroid hormone stimulates Na/K-ATPase activity in skeletal muscle cells [9]. Second, increased physical activity, stress, and thyrotoxicosis all increase catecholamine production. Increased adrenergic response increases Na/K-ATPase activity [8, 10]. Third, hyperinsulinemia leads to worsening of the intracellular shift of potassium in skeletal muscle cells. This explains why the consumption of heavy carbohydrate meals may promote a paralytic attack [11, 12]. Only 2% of patients with hyperthyroidism develop paralysis and as was illustrated by our patient, this proposed model explaining increased activity of Na/K-ATPase may not fully explain the mechanism for HTPP [4]. Although our patient had uncontrolled hyperthyroidism, he gave no history of any precipitating factor such as intense activity or intake of a high carbohydrate meal.

Genetic studies have demonstrated that various gain-of-function and loss-of-function mutations are associated with periodic paralysis [13]. Mutations in Kir2.6 (a potassium ion channel) are seen in up to one-third of patients with HTPP [14]. It is possible that the identification of further gene mutations could help to identify individuals with hyperthyroidism that are at risk for periodic paralysis.

The treatment of an acute presentation of HTPP starts with intravenous or oral supplementation of potassium chloride and use of intravenous or oral propranolol. A retrospective study had demonstrated that patients who received intravenous potassium recovered faster than those who received oral supplementation [15]. However, it should be noted that as potassium is shifted back into the intracellular compartment, excessive potassium replacement may result in rebound hyperkalemia [4]. Propranolol is believed to interfere with the Na/K-ATPase channel and its administration not only mitigates thyrotoxic symptoms, but also addresses the concerns of rebound hyperkalemia and helps to raise the serum levels of potassium and phosphate [1].

Ultimately, the management of HTPP depends upon adequate treatment of the patient’s hyperthyroidism. Recurrent paralytic episodes can be prevented with strict medication compliance. Patients should avoid precipitating factors such as heavy carbohydrate meals, high-salt diet, alcohol use, and significant physical activity [1]. The use of a non-selective beta-blocker like propranolol is useful until euthyroid status is achieved [4].

The management of concurrent conditions such as asthma and hyperthyroidism poses a dual risk. The treatment of asthma with beta-2 agonist medications may lead to intracellular shift of potassium. This can precipitate paralysis whereas the treatment of HTPP with beta-blockers may lead to worsening of pulmonary symptoms.

## Conclusion

In conclusion, this case highlights the challenges faced in the diagnosis and treatment of HTPP in a young African-American male who suffered from Graves’ disease as well as pulmonary asthma. HTPP remains a rare entity in non-Asian populations and early diagnosis helps to prevent adverse outcomes. Good medication compliance and maintenance of euthyroid status in patients with hyperthyroidism is paramount to prevent recurrent episodes of a life-threatening condition such as HTPP.

## Data Availability

Data sharing not applicable to this article as no datasets were generated or analyzed.

## Abbreviations

HTPP: Hypokalemic thyrotoxic periodic paralysis
TSH: Thyroid-stimulating hormone
Na/K-ATPase: Sodium-potassium-adenosine triphosphate
